# Supplementary material for: Anticholinesterase and Serotoninergic Evaluation of Benzimidazole–Carboxamides as Potential Multifunctional Agents for the Treatment of Alzheimer’s Disease
Source: Pharmaceutics. 2023 Aug 19;15(8):2159. doi: 10.3390/pharmaceutics15082159 (PMC10459044; doi:10.3390/pharmaceutics15082159)
Supplement: Supplementary file 1 [file pharmaceutics-15-02159-s001.zip › pharmaceutics-2483960-supplementary.pdf]

Synthesis of Benzimidazole–Carboxamides.

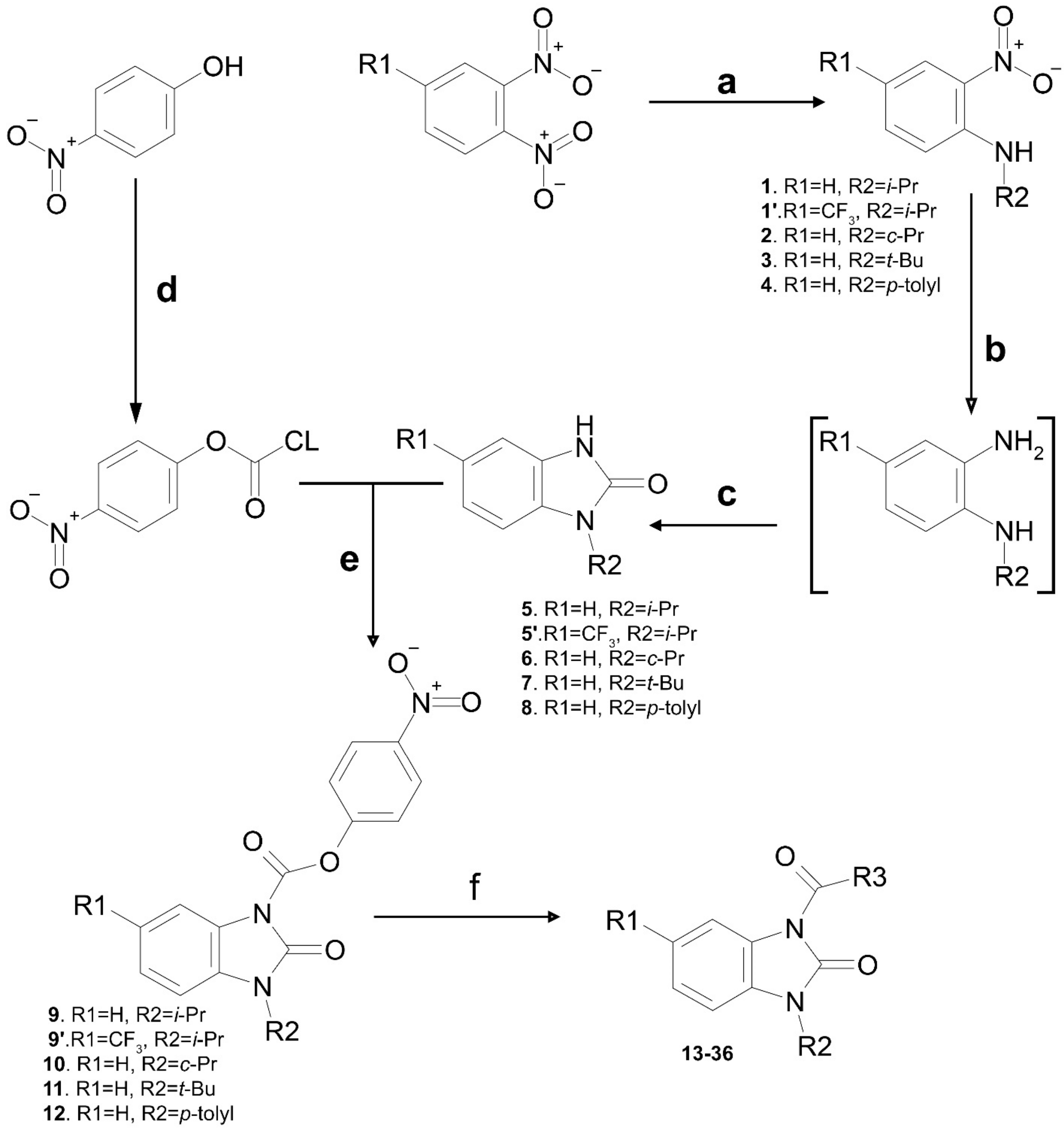

Figure S1. The general scheme for the synthesis of substituted 1,3-dihydro-2-oxo-1*H*-benzimidazol-2-ones. Reagents: **a** — NH_2-R2, EtOH, ref; **b** — THF, Pd/C; **c** — $ClCOOCCl_3$, TEA, 5 ºC; **d** — $ClCOOCCl_3$, DCM, TEA, 5ºC; **e** — toluene, TEA, ref; **f** — NH_2-R3, DCM, TEA.

Table S1. The structures of the intermediate (**1-12**) and target (**13-36**) compounds

Intermediate compounds **1-4**	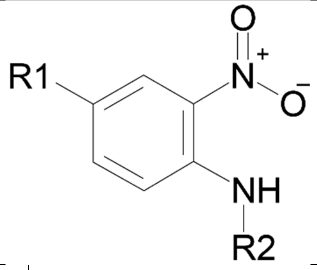	
**No. of compound**	**R1**	**R2**
**1**	H	*i*-Pr
**1'**	CF_3	*i*-Pr
**2**	H	*c*-Pr
**3**	H	*t*-Bu
**4**	H	*p*-tolyl
Intermediate compounds **5-8**	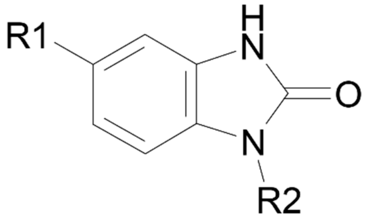	
**No. of compound**	**R1**	**R2**
**5**	H	*i*-Pr
**5'**	CF_3	*i*-Pr
**6**	H	*c*-Pr
**7**	H	*t*-Bu
**8**	H	*p*-tolyl
Intermediate compounds **9-12**	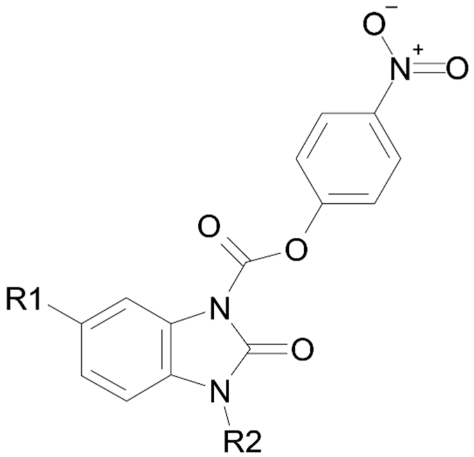	
**No. of compound**	**R1**	**R2**
**9**	H	*i*-Pr
**9'**	CF_3	*i*-Pr
**10**	H	*c*-Pr
**11**	H	*t*-Bu
**12**	H	*p*-tolyl

Target compounds **13-36**	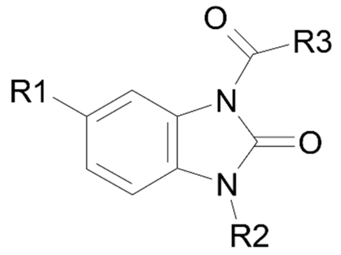		
**No. of compound**	**R₁**	**R₂**	**R₃**
**13**	H	*t*-Bu	N,N-(diethylamino)ethyl
**14**	H	*p*-tolyl	8-methyl-8-azabicyclo[3.2.1]octan-6-yl
**15 (BIMU-8)**	H	*i*-Pr	8-methyl-8-azabicyclo[3.2.1]octan-6-yl
**16**	CF_3	*i*-Pr	8-methyl-8-azabicyclo[3.2.1]octan-6-yl
**17**	H	*i*-Pr	3,5-dimethyltricyclo[3.3.1.1]-dec-1-yl
**18**	H	*i*-Pr	1-adamantan-1-yl
**19**	H	*i*-Pr	2-adamantan-1-yl
**20**	H	*i*-Pr	piperazin-1-yl
**21**	H	*i*-Pr	3-carboxamide-piperidin-1-yl
**22**	H	*i*-Pr	N,N-(diethylamino)ethyl
**23**	CF_3	*i*-Pr	N,N-(diethylamino)ethyl
**24**	H	*i*-Pr	[(4-chlorophenyl)(phenyl)methyl]-piperazin-1-yl
**25**	H	*c*-Pr	8-methyl-8-azabicyclo[3.2.1]octan-6-yl
**26**	H	*c*-Pr	piperazin-1-yl
**27**	H	*c*-Pr	N,N-(diethylamino)ethyl
**28**	H	*c*-Pr	N,N-(dimethylamino)ethyl
**29**	H	*c*-Pr	3-carboxamide-piperidin-1-yl
**30**	H	*c*-Pr	4-(pyrrolidin-1-yl)piperidin-1-yl
**31**	H	*c*-Pr	3,5-dimethyltricyclo[3.3.1.1]-dec-1-yl
**32**	H	*c*-Pr	2-adamantan-1-yl
**33**	H	*c*-Pr	[(4-chlorophenyl)(phenyl)methyl]-piperazin-1-yl
**34**	H	*t*-Bu	[(4,4-difluorophenyl)(phenyl)methyl]piperazin-1-yl
**35**	H	*t*-Bu	8-methyl-8-azabicyclo[3.2.1]octan-6-yl
**36**	H	*p*-tolyl	[(4-chlorophenyl)(phenyl)methyl]-piperazin-1-yl

2-nitro-N-(propane-2-yl)aniline (1). Compound 1 was obtained by boiling 1,2-dinitrobenzene (4-trifluoromethyl-1,2-dinitrobenzene) (1 equiv) with i-propylamine (4 equiv) in ethanol (50 ml) for 10 hours. At the end of the exposure, the solvent was evaporated. The residue from evaporation was separated with column chromatography using the dry method (ethyl acetate/hexane, 9:1), and an orange-colored oil was obtained in a 85% yield: ^{1}H NMR (400 MHz, DMSO) δ 8.06 (dd, 1H), 7.88 (d, J = 8.7, 1.3 Hz, 1H), 7.53 (t, J = 11.2, 4.1 Hz, 1H), 7.07 (d, J = 8.7 Hz, 1H), 6.67 (t, J = 11.1, 4.0 Hz, 1H), 3.92 (dq, 1H) 1.25 (d, 6H).

Products **2-4** were obtained similarly.

N-cyclopropyl-2-nitroaniline (2) was obtained from *o*-dinitrobenzene (1 equiv) and cyclopropanamine (4 equiv) in ethanol (50 ml). The reaction mass was heated to 60 ℃ and left with stirring for 72 hours. Compound **2** was obtained in a 70% yield as orange-colored oil: ^{1}H NMR

(400 MHz, CDCl3): δ 8.20 (d, 1H); 7.61-7.42 (m, 2H); 7.30 (dd, 1H); 6.72 (m, 1H); 2.60 (m, 1H); 0.81-1.01 (m, 2H); 0.60-0.72 (m, 2H).

N-tert-butyl-2-nitroaniline (3) was obtained from *o*-dinitrobenzene (1 equiv) and tert-butylamine (2.5 equiv) in ethanol (50 ml). The reaction mass was heated to 50℃ with stirring for 48 hours. Compound 3 was obtained in an 82% yield as a fluid orange oil: ^{1}H NMR (400 MHz, DMSO) δ 8.25 (s, 1H), 8.07 (dd, J = 8.7, 1.3 Hz, 1H), 7.50 (dd, J = 11.2, 4.1 Hz, 1H), 7.26 (d, J = 8.7 Hz, 1H), 6.66 (dd, J = 11.1, 4.0 Hz, 1H), 1.45 (s, 9H).

4-methyl-N-phenyl-2-nitroaniline (4) was obtained from *o*-dinitrobenzene (1 equiv) and *p*-aminotoluidine (1 equiv) in ethanol (50 ml) with the addition of triethylamine (TEA) (1.5 eq). Compound **4** was obtained in a 63% yield as orange-colored oil, which solidified later: ^{1}H NMR (400 MHz, DMSO): δ 9.36 (s, 1H), 8.10 (dd, J = 8.6, 1.4 Hz, 1H), 7.53 – 7.42 (m, 1H), 7.27 – 7.17 (m, 4H), 7.12 – 7.06 (m, 1H), 6.83 (ddd, J = 8.3, 7.1, 1.0 Hz, 1H), 2.32 (s, 3H).

1-(propane-2-yl)-1,3-dihydro-2*H*-benzimidazole-2-one (5).

The obtained nitroamine **1** (1 equiv) was reducted with hydrogen in a solution of anhydrous tetrahydrofuran (100 ml) in the presence of a catalyst (10% palladium on a carbon matrix, Type 487). The reaction was carried out at an excess pressure of 6 atm and a temperature of 50 ° C for 6 hours. At the end of the reaction, the catalyst was filtered and the obtained diamine was used in the next stage without segregation. The reaction mass was treated with trichloromethyl chloroformate (1.05 equiv) in the presence of TEA (2.5 equiv) at a temperature of 0-5 ° C, then kept at room temperature for about 12 hours. The resulting precipitate was filtered, the filtrate was evaporated and the remainder was dissolved in 100 ml of acetonitrile and boiled with 5 g of silica gel (KSKG, 0.125-0.2 mm) for 1 hour to remove impurities. Then, the suspension was cooled, the silica gel was filtered and the filtrate was evaporated. Product **5** was received in an 85% yield. ^{1}H NMR (400 MHz, DMSO) δ 10.81 (s, 1H), 7.22 (d, J = 7.3 Hz, 1H), 6.96 (d, J = 5.4 Hz, 3H), 4.56 (dt, 1H), 1.42 (d, 6H).

Products **6-8** were obtained similarly.

1-(cyclopropyl)-1,3-dihydro-2*H*-benzimidazole-2-one (6) was derived from N-cyclopropyl-2-nitroaniline (2) (1 equiv). Compound **6** was obtained in an 86% yield: ^{1}H NMR (400 MHz, DMSO) δ 10.81 (s, 1H), 7.22 (d, J = 7.3 Hz, 1H), 6.96 (d, J = 5.4 Hz, 3H), 4.56 (dt, 1H), 0.78 (m, 2H), 0.71 (m, 2H).

1-(tert-butyl)-1,3-dihydro-2*H*-benzimidazole-2-one (7) was derived from N-tert-butyl-2-nitroaniline (3) (1 equiv). Compound **7** was obtained in a 98% yield: ^{1}H NMR (400 MHz, DMSO) δ 10.75 (s, 1H), 7.38 (d, J = 7.3 Hz, 1H), 6.91 (q, J = 5.4 Hz, 3H), 1.68 (s, 9H).

1-(4-methylphenyl)-1,3-dihydro-2H-benzimidazole-2-one (8) was obtained from the solution of 4-methyl-N-phenyl-2-nitroaniline (4) (1 equiv). Compound **8** was obtained in a 44% yield: ^{1}H NMR (400 MHz, DMSO) δ 9.36 (s, 1H), 8.10 (dd, J = 8.6, 1.4 Hz, 1H), 7.53 – 7.42 (m, 1H), 7.27 – 7.17 (m, 4H), 7.12 – 7.06 (m, 1H), 6.83 (ddd, J = 8.3, 7.1, 1.0 Hz, 1H), 2.32 (s, 3H).

***P*-nitrophenyl chloroformate** was obtained by the processing of *p*-nitrophenol (1 equiv) with trichloromethyl chloroformate (1.4 equiv) in the presence of TEA (1 equiv) in 50 ml of dichloromethane at 0-5 ° C, then it was kept at room temperature for about 12 hours. The resulting precipitate was filtered, the filtrate was evaporated, the remainder was dissolved in 100 ml of the mixture tetrahydrofuran/toluene (2:1), the formed precipitate was filtered, the filtrate was evaporated and the procedure was repeated twice. *P*-nitrophenyl chloroformate was obtained in an

86% yield as colorless flakes: ^{1}H NMR (400 MHz, DMSO-d6) δ 8.28 (d, J = 7.6 Hz, 2H), 7.33 (d, J = 7.5 Hz, 2H).

4-nitrophenyl-1-(propane-2-yl)-2-oxo-2,3-dihydro-1*H*-benzimidazole-1-carboxylate (9) was obtained by boiling compound 5 (1 equiv) with *p*-nitrophenyl chloroformate (1.7 equiv) in the presence of TEA (5 equiv) in 50 ml of toluene for 2 hours in an inert argon atmosphere, which was pumped from a balloon. The reaction product was given with filtration in a 36% yield: ^{1}H NMR (400 MHz, DMSO) δ 8.38 (d, J = 8.8 Hz, 2H), 7.82 (d, J = 7.9 Hz, 1H), 7.69 (d, J = 8.8 Hz, 2H), 7.45 (d, J = 7.8 Hz, 1H), 7.22 (dt, J = 42.1, 7.6 Hz, 2H), 4.67 (dt, J = 13.4, 6.6 Hz, 1H), 1.50 (d, J = 6.8 Hz, 6H).

Products **10-12** were obtained similarly.

4-nitrophenyl-1-(cyclopropyl)-2-oxo-2,3-dihydro-1*H*-benzimidazole-1-carboxylate (10) was obtained from cyclopropyl-1,3-dihydro-2*H*-benzimidazole-2-one (**6**) (1 equiv) and *p*-nitrophenyl chloroformate (1.2 equiv) in toluene (50 ml) with the addition of TEA (1.5 equiv). Compound **10** was obtained in a 33% yield as a white crystalline precipitate: ^{1}H NMR (400 MHz, DMSO) δ 8.38 (d, J = 8.8 Hz, 2H), 7.82 (d, J = 7.9 Hz, 1H), 7.69 (d, J = 8.8 Hz, 2H), 7.45 (d, J = 7.8 Hz, 1H), 7.22 (dt, J = 42.1, 7.6 Hz, 2H), 4.67 (dt, J = 13.4, 6.6 Hz, 1H), 0.77 (m, 2H), 0.73 (m, 2H).

4-nitrophenyl-1-(tert-butyl)-2-oxo-2,3-dihydro-1*H*-benzimidazole-1-carboxylate (11) was derived from tert-butyl-1,3-dihydro-2*H*-benzimidazole-2-one (**7**) (1 equiv) and *p*-nitrophenyl chloroformate (1.2 equiv) in toluene (50 ml) with the addition of TEA (1.5 equiv). Compound **11** was obtained in a 27% yield as a crystalline precipitate of a light pink, almost white color: ^{1}H NMR (400 MHz, DMSO) δ 8.38 (dd, J = 9.7, 2.7 Hz, 2H), 7.85 (d, J = 7.9 Hz, 1H), 7.73 – 7.57 (m, 3H), 7.25 – 7.09 (m, 2H), 1.75 (s, 9H).

4-nitrophenyl-1-(methylphenyl)-2-oxo-2,3-dihydro-1*H*-benzimidazole-1-carboxylate (12) was derived from 1-(4-methylphenyl)-1,3-dihydro-2*H*-benzimidazole-2-one (**8**) (1 equiv) and *p*-nitrophenyl chloroformate (1.2 equiv) in toluene (50 ml) with the addition of TEA (1.5 equiv). Compound **12** was obtained in a 32% yield: ^{1}H NMR (400 MHz, DMSO) δ 8.40 (d, J = 9.1 Hz, 12H), 7.94 – 7.86 (m, 6H), 7.71 (d, J = 9.1 Hz, 12H), 7.45 (s, 29H), 7.26 (dd, J = 6.1, 2.5 Hz, 13H), 7.09 – 7.03 (m, 2H), 7.01 – 6.89 (m, 8H), 2.42 (s, 17H).

Target compounds **13-36** were obtained with the following method:

N-[2-(diethylamino)ethyl]-2-oxo-3-(tert-butyl)-2,3-dihydro-1*H*-benzimidazole-1-carboxamide hydrochloride (13). Compound **11** (1 equiv) was dissolved in 50 ml of methylene chloride and mixed with N,N-diethylethane-1,2-diamine (1.2 equiv) and TEA (3 equiv) at room temperature for 12 hours. At the end of the exposure, the reaction mass was washed three times with 50 ml of water, the organic layer was separated, dried over 5 g of magnesium sulfate (anhydrous) for 30 minutes, magnesium sulfate was filtered and the filtrate was concentrated. The residue from evaporation was recrystallized from 10 ml of acetonitrile. The obtained oily product was dissolved in diethyl ether and dioxane hydrochloride was added using dripping; product **13** precipitated. Then, it was filtered and dried. Compound **13** was obtained in an 82% yield as white solids: ^{1}H NMR (400 MHz, DMSO) δ 10.34 (d,1H), 9.08 (d, 1H), 8.15 (dd, 1H), 7.63 (dd, 1H), 7.19 (d, 1H), 3.69 (2H), 2.47 (2H), 1.74 (s, 9H), 1.25 (s, 6H). HPLC-MS m/z calculated for: $C_{18}H_{28}N_4O_2$ HCl $[M+H]^+$ 369.2052; found: 369.1953. ^{13}C NMR (100 MHz, DMSO-*d*$_6$) δ 153.05, 151.90, 129.55, 126.95, 123.98, 122.62, 113.43, 110.41, 58.21, 49.48, 47.15, 34.66, 24.94, 10.11.

Products **14-36** were obtained similarly.

N-(8-methyl-8-azabicyclo[3.2.1]octane-6-yl)-1-carbonyl-3-(4-methylphenyl)-2,3-dihydro-1*H*-benzimidazole-2-one (14) was obtained from compound **12** (1 equiv) and aminotropin (1.2 equiv) with the addition of TEA (3 equiv). Compound **14** was obtained in a 71% yield as white solids: ^{1}H NMR (400 MHz, DMSO-d6) δ 8.71 (d, J = 10.4 Hz, 1H), 8.21 (dd, J = 7.4, 1.5 Hz, 1H), 7.77 (dd, J = 7.4, 1.6 Hz, 1H), 7.48 (td, J = 7.5, 1.5 Hz, 1H), 7.31 (d, J = 7.6 Hz, 2H), 7.19 (td, J = 7.5, 1.5 Hz, 1H), 7.12 (d, J = 6.9 Hz, 2H), 3.81 (dt, J = 10.4, 4.0 Hz, 1H), 3.01 (td, J = 3.0, 1.4 Hz, 2H), 2.45 (t, J = 1.5 Hz, 3H), 2.39 (d, J = 0.9 Hz, 3H), 2.05 – 1.93 (m, 2H), 1.89 (ddd, J = 12.4, 4.0, 3.2 Hz, 2H), 1.80 (ddd, J = 12.4, 4.0, 3.2 Hz, 2H), 1.66 (ddd, J = 10.3, 5.9, 2.3 Hz, 2H). HPLC-MS m/z calculated for: $C_{23}H_{26}N_4O_2$ 391.2129 $[M+H]^+$; found: 391.1129.

N-(8-methyl-8-azabicyclo[3.2.1]octane-6-yl)-1-carbonyl-3-(propane-2-yl)-2,3-dihydro-1*H*-benzimidazole-2-one (15) was obtained from compound **9** (1 equiv) and aminotropin (1.2 equiv) with the addition of TEA (3 equiv). Compound **15** was obtained in a 45% yield as white solids: ^{1}H NMR (400 MHz, DMSO) δ 9.29 (d, J = 7.6 Hz, 1H), 8.08 (d, J = 8.0 Hz, 1H), 7.44 (d, J = 7.8 Hz, 1H), 7.20 (t, J = 7.7 Hz, 1H), 7.14 (t, J = 7.7 Hz, 1H), 4.69 (dt, J = 13.9, 6.9 Hz, 1H), 3.99 (q, J = 6.9 Hz, 1H), 3.05 (s, 2H), 2.17 (s, 3H), 2.04 (dd, J = 13.4, 8.6 Hz, 4H), 1.85 (d, J = 8.0 Hz, 2H), 1.60 (d, J = 14.2 Hz, 2H), 1.48 (d, J = 7.0 Hz, 6H). HPLC-MS m/z calculated for: $C_{19}H_{26}N_4O_2$ HCl $[M+H]^+$ 379.1895; found: 379.0899.

N-(8-Methyl-8-azabicyclo[3.2.1]octane-6-yl)-2-oxo-3-(propane-2-yl)-6-(trifluoromethyl)-2,3-dihydro-1*H*-benzimidazole-1-carboxamide hydrochloride (16) was derived from 4-nitrophenyl-1-(propane-2-yl)-2-oxo-6-(trifluoromethyl)-2,3-dihydro-1*H*-benzimidazole-1-carboxylate (1 equiv) and aminotropin (1.2 equiv) with the addition of TEA (3 equiv). Compound **16** was obtained in a 10% yield as white solids: ^{1}H NMR (400 MHz, DMSO) δ 9.17 (d, J = 7.6 Hz, 1H), 8.38 (s, 1H), 7.62 (dd, J = 34.0, 8.4 Hz, 2H), 4.73 (dt, J = 13.9, 6.9 Hz, 1H), 4.01 (d, J = 7.1 Hz, 1H), 3.09 (s, 2H), 2.27 – 1.99 (m, 8H), 1.87 (d, J = 7.9 Hz, 2H), 1.63 (d, J = 14.3 Hz, 2H), 1.48 (dd, J = 20.9, 7.0 Hz, 7H). HPLC-MS m/z calculated for: $C_{20}H_{25}F_3N_4O_2$ $[M+H]^+$ 411.2002; found: 411.1002.

N-(3,5-dimethyltricyclo[3.3.1.1]-dec-1-yl)-3-(propane-2-yl)-2-oxo-2,3-dihydro-1*H*-benzimidazole-1-carboxamide (17) was obtained from compound **9** (1 equiv) and memantine (1.1 equiv) with the addition of TEA (3 equiv). Compound **17** was obtained in a 71% yield as white solids: ^{1}H NMR (400 MHz, DMSO) δ 8.77 (s, 1H), 8.06 (d, J = 8.0 Hz, 1H), 7.43 (d, J = 7.9 Hz, 1H), 7.16 (dt, J = 30.0, 7.7 Hz, 2H), 4.64 (hept, J = 6.8 Hz, 1H), 2.14 (s, 1H), 1.87 (s, 2H), 1.68 (dd, J = 25.6, 11.6 Hz, 4H), 1.47 (d, J = 6.9 Hz, 6H), 1.33 (dd, J = 33.3, 12.1 Hz, 4H), 1.20 – 1.11 (m, 2H), 0.85 (s, 6H). HPLC-MS m/z calculated for: $C_{23}H_{31}N_3O_2$ $[M+H]^+$ 380.2332; found: 380.1332.

N-(1-adamantane-1-yl)-3-(propane-2-yl)-2-oxo-2,3-dihydro-1*H*-benzimidazole-1-carboxamide (18) was obtained from compound **9** (1 equiv) and 1-adamantanamine (1.1 equiv) with the addition of TEA (3 equiv). Compound **18** was obtained in a 68% yield as white solids: ^{1}H NMR (400 MHz, DMSO) δ 8.75 (s, 1H), 8.08 (d, J = 7.7 Hz, 1H), 7.42 (d, J = 7.8 Hz, 1H), 7.16 (dt, J = 29.3, 7.5 Hz, 2H), 4.65 (dt, J = 13.8, 6.9 Hz, 1H), 2.05 (d, J = 14.7 Hz, 9H), 1.67 (s, 6H), 1.48 (d, J = 6.9 Hz, 6H). HPLC-MS m/z calculated for: $C_{21}H_{27}N_3O_2$ $[M+H]^+$ 354.2176; found: 354.1176.

N-(2-adamantane-1-yl)-3-(propane-2-yl)-2-oxo-2,3-dihydro-1*H*-benzimidazole-1-carboxamide (19) was obtained from compound **9** (1 equiv) and 2-adamantanamine (1.1 equiv) with the addition of TEA (3 equiv). Compound **19** was obtained in a 43% yield as white solids: ^{1}H NMR (400 MHz, DMSO) δ 9.31 (d, J = 7.3 Hz, 1H), 8.09 (d, J = 7.5 Hz, 1H), 7.45 (d, J = 7.3 Hz, 1H), 7.18 (dd, J = 18.5, 7.4 Hz, 2H), 4.82 – 4.56 (m, 1H), 4.02 (s, 1H), 1.96 – 1.60 (m, 14H), 1.50

(d, J = 6.2 Hz, 6H). HPLC-MS m/z calculated for: $C_{21}H_{27}N_3O_2$ $[M+H]^+$ 354.2176; found: 354.1176.

1-(piperazine-1-carbonyl)-3-(propane-2-yl)-1,3-dihydro-2*H*-benzimidazole-2-oh hydrochloride (20) was obtained from compound **9** (1 equiv) and piperazine (1.2 equiv) with the addition of TEA (3 equiv). Compound **15** was obtained in a 60% yield as white solids: ^{1}H NMR (400 MHz, DMSO) δ 9.62 (s, 2H), 7.40 (d, J = 7.7 Hz, 1H), 7.33 – 7.24 (m, 1H), 7.13 (dtd, J = 24.8, 7.6, 1.0 Hz, 2H), 4.68 – 4.51 (m, 1H), 3.67 (d, J = 83.7 Hz, 4H), 3.31 – 3.09 (m, 4H), 1.47 (d, J = 6.9 Hz, 6H). HPLC-MS m/z calculated for: $C_{15}H_{20}N_4O_2$ HCl $[M+H]^+$ 325.1425; found: 325.0425.

1-[2-oxo-3-(propane-2-yl)-2,3-dihydro-1*H*-benzimidazole-1-carbonyl]piperidine-3-carboxamide (21) was obtained from compound **9** (1 equiv) and piperidine-3-carboxamide (1.2 equiv) with the addition of TEA (3 equiv). Compound **21** was obtained in a 19% yield as white solids: ^{1}H NMR (400 MHz, DMSO) δ 7.48 – 7.28 (m, 2H), 7.26 – 7.02 (m, 3H), 6.84 (s, 1H), 4.60 (dt, J = 13.9, 6.9 Hz, 1H), 4.26 (s, 1H), 3.68 (s, 1H), 3.06 (s, 2H), 2.39 (t, J = 11.0 Hz, 1H), 1.58 (dd, J = 81.8, 35.4 Hz, 10H). HPLC-MS m/z calculated for: $C_{17}H_{22}N_4O_3$ $[M+H]^+$ 331.1764; found: 331.0764.

N-[2-(diethylamino)ethyl]-2-oxo-3-(propane-2-yl)-2,3-dihydro-1*H*-benzimidazole-1-carboxamide hydrochloride (22) was obtained from compound 9 (1 equiv) and N,N-diethylethane-1,2-diamine (1.2 equiv) with the addition of TEA (3 equiv). Compound **22** was obtained in an 80% yield as white solids: ^{1}H NMR (400 MHz, DMSO) δ 10.60 (s, 1H), 8.96 (t, J = 5.8 Hz, 1H), 8.14 – 7.99 (m, 1H), 7.44 (d, J = 7.8 Hz, 1H), 7.29 – 7.06 (m, 2H), 4.76 – 4.58 (m, 1H), 3.71 (d, J = 6.1 Hz, 2H), 3.30 – 3.01 (m, 6H), 1.49 (d, J = 6.9 Hz, 6H), 1.25 (t, J = 7.1 Hz, 6H). HPLC-MS m/z calculated for: $C_{17}H_{26}N_4O_2$ HCl $[M+H]^+$ 355.1895; found: 355.0895.

N-[2-(diethylamino)ethyl]-2-oxo-3-(propane-2-yl)-6-(trifluoromethyl)-2,3-dihydro-1*H*-benzimidazole-1-carboxamide hydrochloride (23) was derived from 4-nitrophenyl-1-(propane-2-yl)-2-oxo-6-(trifluoromethyl)-2,3-dihydro-1*H*-benzimidazole-1-carboxylate (1 equiv) and N,N-diethylethane-1,2-diamine (1.2 equiv) with the addition of TEA (3 equiv). Compound **23** was obtained in a 19% yield as white solids: ^{1}H NMR (400 MHz, DMSO) δ 10.22 (s, 1H), 8.90 (t, J = 5.8 Hz, 1H), 8.36 (d, J = 1.0 Hz, 1H), 7.63 (dd, J = 26.6, 8.4 Hz, 2H), 4.88 – 4.62 (m, 1H), 3.70 (s, 2H), 3.21 (d, J = 30.2 Hz, 6H), 1.51 (d, J = 6.9 Hz, 6H), 1.24 (t, J = 6.4 Hz, 6H). HPLC-MS m/z calculated for: $C_{18}H_{25}F_3N_4O_2$ HCl $[M+H]^+$ 423.1769; found: 423.0768.

[(4-chlorophenyl)(phenyl)methyl]piperazine-1-carbonyl-3-(propane-2-yl)-2,3-dihydro-1*H*-1,3-benzimidazole-2-one hydrochloride (24) was obtained from compound **9** (1 equiv) and 1-[(4-chlorophenyl)(phenyl)methyl]piperazine (1 equiv) with the addition of TEA (3 equiv). Compound **24** was obtained in a 27% yield as white solids: ^{1}H NMR (400 MHz, CDCl3) δ 7.65 (d, J = 8.0 Hz, 4H), 7.50 – 7.35 (m, 6H), 7.22 – 7.12 (m, 3H), 4.79 (s, 1H), 4.64 (dt, J = 14.0, 7.0 Hz, 1H), 3.99 (d, J = 80.3 Hz, 4H), 3.36 (s, 4H), 1.55 (dd, J = 9.7, 6.3 Hz, 6H). HPLC-MS m/z calculated for: $C_{28}H_{29}ClN_4O_2$ $[M+H]^+$ 489.2051; found: 489.1051.

N-(8-methyl-8-azabicyclo[3.2.1]octane-3-yl)-2-oxo-3-(cyclopropyl)-2,3-dihydro-1*H*-benzimidazole-1-carboxamide hydrochloride (25) was obtained from compound **10** (1 equiv) and aminotropin (1.2 equiv) with the addition of TEA (3 equiv). Compound **25** was obtained in a 38% yield as white solids: ^{1}H NMR (400 MHz, DMSO) δ 9.24 (d, J = 7.6 Hz, 1H), 8.03 (d, J = 7.8 Hz, 1H), 7.23 (ddd, J = 35.5, 21.1, 7.6 Hz, 3H), 3.99 (dd, J = 13.9, 6.8 Hz, 1H), 3.06 (s, 2H), 2.96 (tt, J = 6.9, 3.7 Hz, 1H), 2.18 (s, 3H), 2.15 – 1.97 (m, 4H), 1.87 (t, J = 7.1 Hz, 2H), 1.60 (d, J =

14.1 Hz, 2H), 1.09 (q, J = 7.1 Hz, 2H), 1.01 – 0.88 (m, 2H). HPLC-MS m/z calculated for: $C_{19}H_{24}N_4O_2$ $[M+H]^+$ 341.1972; found: 341.0972.

1-(piperazine-1-carbonyl)-3-(cyclopropyl)-1,3-dihydro-2*H*-benzimidazole-2-one hydrochloride (26) was obtained from compound **10** (1 equiv) and piperazine (1.1 equiv) with the addition of TEA (3 equiv). Compound **26** was obtained in a 12% yield as white solids: ^{1}H NMR (400 MHz, DMSO) 7.27 (t, J = 6.6 Hz, 2H), 7.20 (t, J = 7.7 Hz, 1H), 7.12 (t, J = 7.7 Hz, 1H), 3.76 (s, 4H), 3.21 (s, 4H), 3.12 – 3.01 (m, 1H), 2.96 – 2.85 (m, 1H), 1.28 – 1.16 (m, 2H), 1.06 (q, J = 6.9 Hz, 2H), 0.96 – 0.87 (m, 2H). HPLC-MS m/z calculated for: $C_{15}H_{18}N_4O_2$ HCl $[M+H]^+$ 323.1269; found: 323.0269.

N-[2-(diethylamino)ethyl]-2-oxo-3-(cyclopropyl)-2,3-dihydro-1*H*-benzimidazole-1-carboxamide hydrochloride (27) was obtained from compound **10** (1 equiv) and N,N-diethylethane-1,2-diamine (1.2 equiv) with the addition of TEA (3 equiv). Compound **27** was obtained in a 78% yield as white solids: ^{1}H NMR (400 MHz, DMSO) δ 10.46 (s, 1H), 8.91 (t, J = 5.8 Hz, 1H), 8.02 (d, J = 7.6 Hz, 1H), 7.38 – 7.30 (m, 1H), 7.26 (td, J = 7.7, 1.0 Hz, 1H), 7.16 (td, J = 7.9, 1.2 Hz, 1H), 3.70 (d, J = 5.4 Hz, 2H), 3.20 (d, J = 25.6 Hz, 6H), 2.96 (tt, J = 7.0, 3.7 Hz, 1H), 1.24 (t, J = 7.0 Hz, 6H), 1.14 – 1.05 (m, 2H), 0.97 – 0.87 (m, 2H). HPLC-MS m/z calculated for: $C_{17}H_{24}N_4O_2$ HCl $[M+H]^+$ 353.1738; found: 353.0738.

N-[2-(dimethylamino)ethyl]-2-oxo-3-(cyclopropyl)-2,3-dihydro-1*H*-benzimidazole-1-carboxamide hydrochloride (28) was obtained from compound **9** (1 equiv) and N,N-dimethylethane-1,2-diamine (1.2 equiv) with the addition of TEA (3 equiv). Compound **28** was obtained in a 62% yield as white solids: ^{1}H NMR (400 MHz, DMSO) δ 10.69 (s, 1H), 8.80 (t, J = 5.9 Hz, 1H), 8.02 (d, J = 7.5 Hz, 1H), 7.40 – 7.28 (m, 1H), 7.25 (td, J = 7.7, 1.1 Hz, 1H), 7.16 (td, J = 7.9, 1.2 Hz, 1H), 3.06 (dd, J = 9.2, 6.7 Hz, 2H), 2.95 (tt, J = 7.0, 3.7 Hz, 1H), 2.72 (s, 6H), 2.06 – 1.85 (m, 2H), 1.15 – 1.02 (m, 2H), 0.99 – 0.85 (m, 2H). HPLC-MS m/z calculated for: $C_{15}H_{20}N_4O_2$ HCl $[M+H]^+$ 325.1425; found: 325.0425.

1-[2-oxo-3-(cyclopropyl)-2,3-dihydro-1*H*-benzimidazole-1-carbonyl]piperidine-3-carboxamide (29) was obtained from compound **10** (1 equiv) and piperidine-3-carboxamide (1.2 equiv) with the addition of TEA (3 equiv). Compound **29** was obtained in a 51% yield as white solids: ^{1}H NMR (400 MHz, DMSO) δ 7.33 (s, 1H), 7.26 (d, J = 7.6 Hz, 1H), 7.22 – 7.13 (m, 2H), 7.09 (t, J = 7.4 Hz, 1H), 6.84 (s, 1H), 4.24 (s, 1H), 3.72 (s, 1H), 3.05 (s, 2H), 2.95 – 2.84 (m, 1H), 2.39 (t, J = 10.9 Hz, 1H), 1.68 (d, J = 67.2 Hz, 4H), 1.04 (d, J = 5.4 Hz, 2H), 0.92 (s, 2H). HPLC-MS m/z calculated for: $C_{17}H_{20}N_4O_3$ $[M+H]^+$ 329.1608; found: 329.0608.

1-cyclopropyl-3-[4-(pyrrolidine-1-yl)-1-carbonyl]-1,3-dihydro-1*H*-benzimidazole-2-oh hydrochloride (30) was obtained from compound **10** (1 equiv) and 4-(pyrrolidine-1-yl)piperidine (1.1 equiv) with added TEA (3 equiv). Compound **30** was obtained in a 69% yield as white solids: ^{1}H NMR (400 MHz, DMSO) δ 11.35 (s, 1H), 7.26 (d, J = 7.4 Hz, 1H), 7.24 – 7.14 (m, 2H), 7.09 (td, J = 7.7, 1.1 Hz, 1H), 4.34 (s, 1H), 3.86 (s, 1H), 3.41 (d, J = 40.0 Hz, 4H), 3.05 (d, J = 6.6 Hz, 4H), 2.91 (dt, J = 10.5, 3.5 Hz, 1H), 2.13 (s, 2H), 1.91 (s, 5H), 1.10 – 1.00 (m, 2H), 0.92 (s, 2H). HPLC-MS m/z calculated for: $C_{20}H_{26}N_4O_2$ HCl $[M+H]^+$ 391.1895; found: 391.0895.

N-(3,5-dimethyltricyclo[3,3,1,1]-dec-1-yl)-3-(cyclopropyl)-2-oxo-2,3-dihydro-1*H*-benzimidazole-1-carboxamide (31) was obtained from compound **10** (1 equiv) and memantine (1.2 equiv) with the addition of TEA (3 eq). Compound **31** was obtained in a 48% yield as white solids: ^{1}H NMR (400 MHz, DMSO) δ 8.71 (s, 1H), 8.02 (d, J = 7.8 Hz, 1H), 7.31 (d, J = 7.6 Hz, 1H), 7.23 (t, J = 7.3 Hz, 1H), 7.14 (t, J = 7.3 Hz, 1H), 2.99 – 2.87 (m, 1H), 2.14 (s, 1H), 1.87 (s, 2H), 1.67 (dd, J = 25.1, 11.6 Hz, 4H), 1.34 (dd, J = 33.2, 12.0 Hz, 4H), 1.16 (s, 2H), 1.06 (q, J =

7.0 Hz, 2H), 0.95 – 0.90 (m, 2H), 0.86 (s, 6H). HPLC-MS m/z calculated for: $C_{23}H_{29}N_3O_2$ $[M+H]^+$ 380.2332; found: 380.1332.

N-(2-adamantane-1-yl)-3-(cyclopropyl)-2-oxo-2,3-dihydro-1*H*-benzimidazole-1-carboxamide (32) was obtained from compound **10** (1 equiv) and 2-adamantanamine (1.2 equiv) with the addition of TEA (3 equiv). Compound **32** was obtained in a 50% yield as white solids: ^{1}H NMR (400 MHz, DMSO) δ 9.26 (d, J = 7.9 Hz, 1H), 8.03 (d, J = 7.8 Hz, 1H), 7.32 (d, J = 7.6 Hz, 1H), 7.24 (t, J = 7.3 Hz, 1H), 7.16 (t, J = 7.3 Hz, 1H), 4.01 (d, J = 7.8 Hz, 1H), 3.02 – 2.88 (m, 1H), 1.94 (s, 2H), 1.83 (d, J = 20.3 Hz, 8H), 1.74 (s, 2H), 1.65 (d, J = 12.3 Hz, 2H), 1.07 (t, J = 5.9 Hz, 2H), 1.00 – 0.90 (m, 2H). HPLC-MS m/z calculated for: $C_{21}H_{25}N_3O_2$ $[M+H]^+$ 352.2019; found: 352.1019.

[(4-Chlorophenyl)(phenyl)methyl]piperazine-1-carbonyl-3-(cyclopropyl)-2,3-dihydro-1*H*-1,3-benzimidazole-2-oh hydrochloride (33) was obtained from compound **10** (1 equiv) and 1-[(4-chlorophenyl)(phenyl)methyl]piperazine (1.2 equiv) with the addition of TEA (3 equiv). Compound **33** was obtained in a 53% yield as white solids: ^{1}H NMR (400 MHz, DMSO) δ 7.46 (dd, J = 19.0, 7.9 Hz, 4H), 7.37 (d, J = 8.5 Hz, 2H), 7.32 (t, J = 7.5 Hz, 2H), 7.23 (t, J = 6.7 Hz, 2H), 7.19 – 7.13 (m, 2H), 7.08 (t, J = 7.2 Hz, 1H), 4.43 (s, 1H), 3.64 (s, 2H), 3.45 (s, 2H), 2.87 (tt, J = 7.0, 3.6 Hz, 1H), 2.39 (s, 4H), 1.02 (q, J = 7.0 Hz, 2H), 0.91 – 0.83 (m, 2H). HPLC-MS m/z calculated for: $C_{28}H_{27}ClN_4O_2$ $[M+H]^+$ 487.1895; found: 487.0895.

[(4,4-difluorophenyl)(phenyl)methyl]piperazine-1-carbonyl-3-(tert-butyl)-2,3-dihydro-1*H*-1,3 benzimidazole-2one (34) was obtained from compound **11** (1 equiv) and 1-[bis(4-fluorophenyl)methyl]piperazine (1.2 equiv) with the addition of TEA (3 equiv). Compound **34** was obtained in a 33% yield as white solids: ^{1}H NMR (400 MHz, DMSO) δ 8.33 (s, 2H), 7.57 – 7.40 (m, 55H), 7.21 – 7.00 (m, 76H), 4.47 (s, 10H), 3.65 (s, 22H), 3.48 – 3.37 (m, 25H), 2.37 (d, J = 28.5 Hz, 31H), 2.23 (s, 9H), 1.69 (s, 96H). HPLC-MS m/z calculated for: $C_{29}H_{30}F_2N_4O_2$ HCl $[M+H]^+$ 525.1863; found: 525.0863.

N-(8-methyl-8-azabicyclo[3.2.1]octane-6-yl)-1-carbonyl-3-(tert-butyl)-2,3-dihydro-1H-benzimidazole-2-one (35) was obtained from compound **11** (1 equiv) and 8-methyl-8-azabicyclo[3.2.1]octane-6-amine (1 equiv) with the addition of TEA (3 equiv). Compound **35** was obtained in a 48% yield as white solids: ^{1}H NMR (400 MHz, DMSO) δ 7.54 (d, J = 2.2 Hz, 10H), 7.08 (s, 33H), 3.47 – 3.17 (m, 65H), 3.09 (s, 8H), 2.08 (d, J = 5.6 Hz, 7H), 1.88 (d, J = 15.4 Hz, 15H), 1.71 (s, 92H), 1.53 (d, J = 15.3 Hz, 16H), 1.23 (s, 9H), 1.01 (d, J = 6.0 Hz, 6H), 0.85 (s, 2H). HPLC-MS m/z calculated for: $C_{20}H_{28}N_4O_2$ HCl $[M+H]^+$ 393.2051; found: 393.1051.

1-[(4-chlorophenyl)(phenyl)methyl]piperazine-1-carbonyl-3-(4-methylphenyl)-2,3-dihydro-1*H*-1,3-benzimidazole-2-one (36) was derived from compound **12** (1 equiv) and 1-[(4-chlorophenyl)(phenyl)methyl]piperazine (1 equiv) with the addition of TEA (3 equiv). Compound **36** was obtained in a 40% yield as white solids: ^{1}H NMR (400 MHz, CDCl3) δ 7.51 (d, J = 7.3 Hz, 4H), 7.42 (d, J = 7.7 Hz, 1H), 7.34 (d, J = 9.9 Hz, 9H), 7.15 (dt, J = 20.1, 7.4 Hz, 2H), 6.97 (d, J = 7.6 Hz, 1H), 4.52 (s, 1H), 3.89 (d, J = 67.4 Hz, 4H), 2.95 (s, 4H), 2.43 (s, 3H). HPLC-MS m/z calculated for: $C_{32}H_{29}ClN_4O_2$ HCl $[M+H]^+$ 573.1818; found: 573.0818.

Anticholinesterase activity of benzimidazole–carboxamides in vitro

A

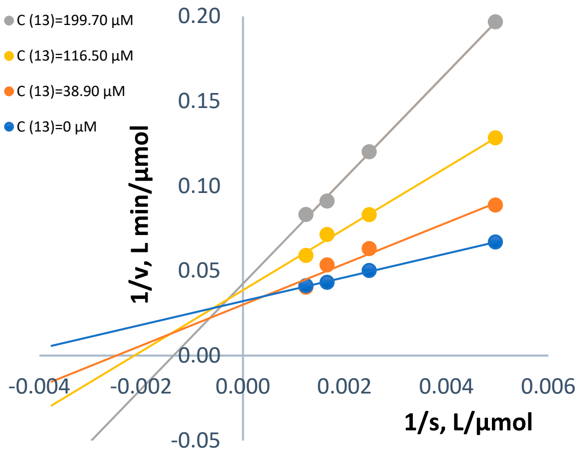

B

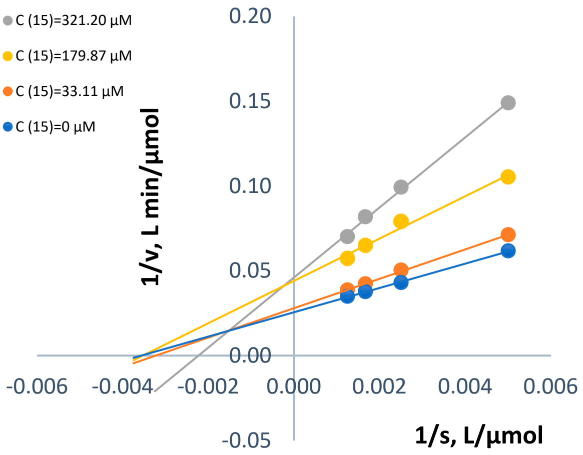

C

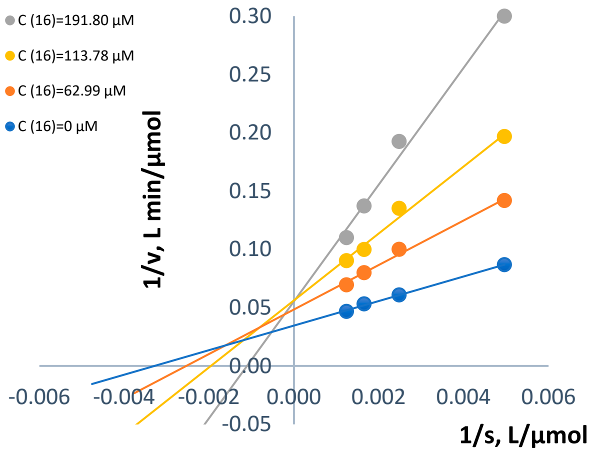

D

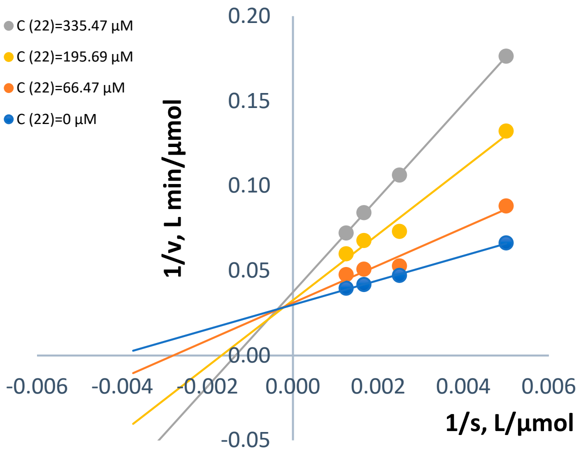

E

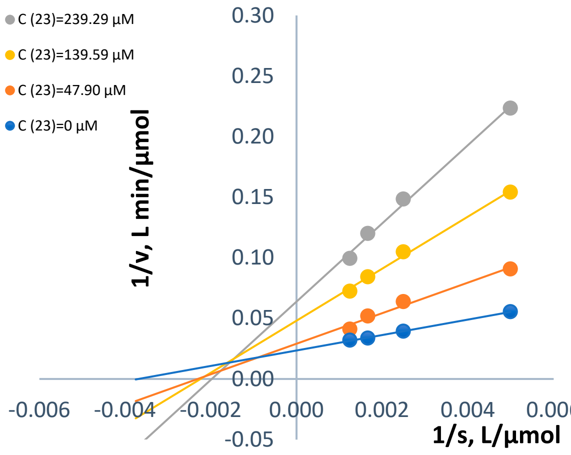

F

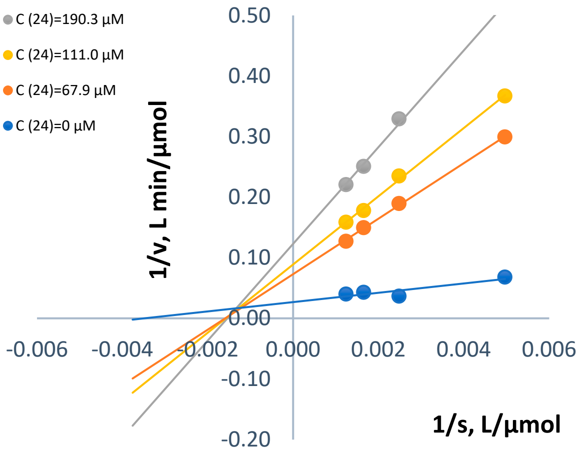

G

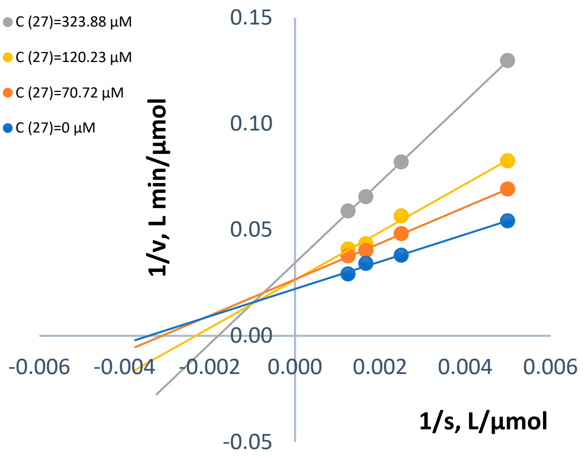

H

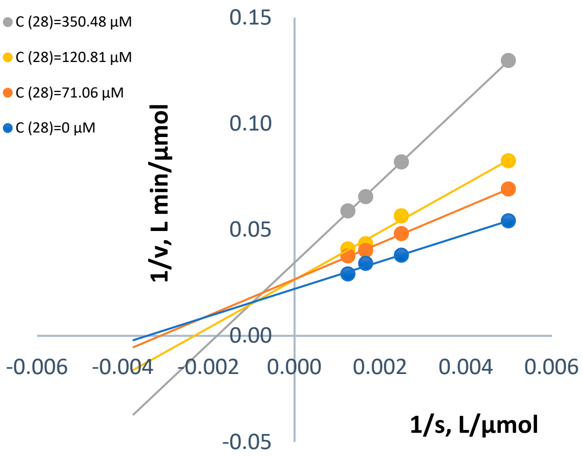

Figure S2. Inhibitory ability of benzimidazole–carboxamides towards AChE according to in vitro data. A – H, Lineweaver–Burk plots 1/v=f (1/[S]) for compounds **13**, **15**, **16**, **22**, **23**, **24**, **27** and **28**, respectively; v, initial reaction rate; [S], substrate concentration; α, thermodynamic cooperativity factor.

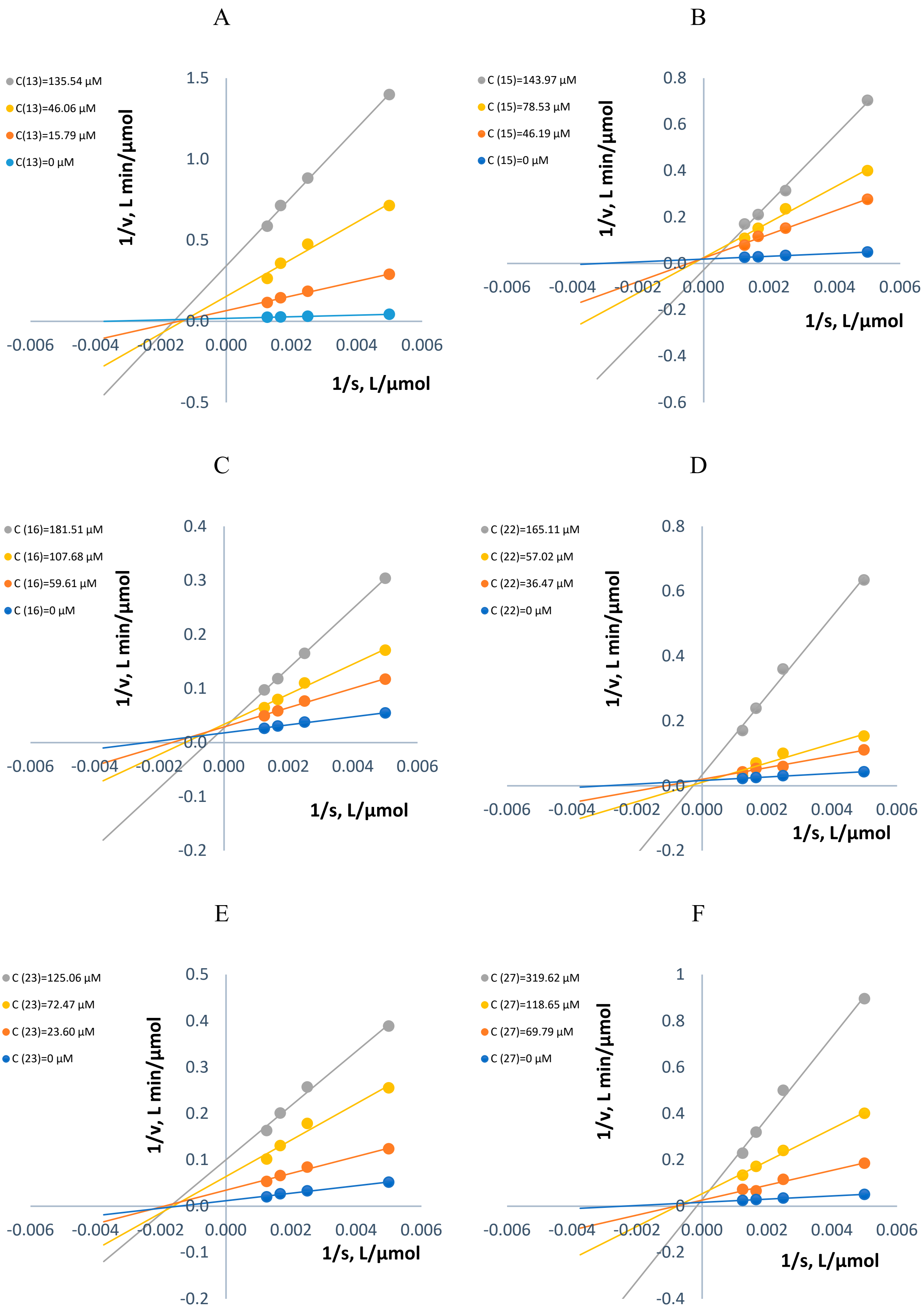
A
C(13)=135.54 μM
C(13)=46.06 μM
C(13)=15.79 μM
C(13)=0 μM
1/v, L min/μmol
1/s, L/μmol
B
C (15)=143.97 μM
C (15)=78.53 μM
C (15)=46.19 μM
C (15)=0 μM
1/v, L min/μmol
1/s, L/μmol
C
C (16)=181.51 μM
C (16)=107.68 μM
C (16)=59.61 μM
C (16)=0 μM
1/v, L min/μmol
1/s, L/μmol
D
C (22)=165.11 μM
C (22)=57.02 μM
C (22)=36.47 μM
C (22)=0 μM
1/v, L min/μmol
1/s, L/μmol
E
C (23)=125.06 μM
C (23)=72.47 μM
C (23)=23.60 μM
C (23)=0 μM
1/v, L min/μmol
1/s, L/μmol
F
C (27)=319.62 μM
C (27)=118.65 μM
C (27)=69.79 μM
C (27)=0 μM
1/v, L min/μmol
1/s, L/μmol

G

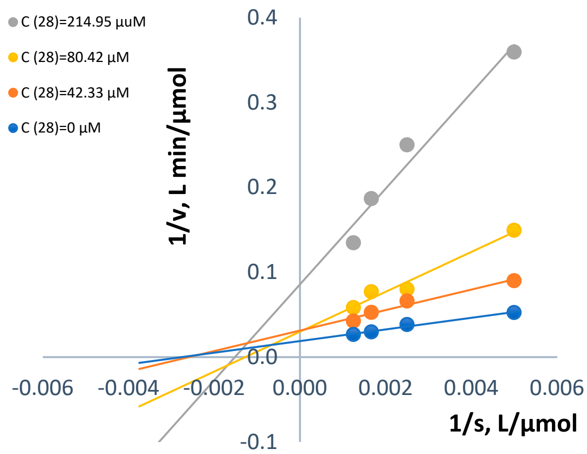

Figure S3. Inhibitory ability of benzimidazole–carboxamides towards BChE according to in vitro data. A – G, Lineweaver–Burk plots 1/v=f (1/[S]) for compounds **13**, **15**, **16**, **22**, **23**, **27** and **28**, respectively; v, initial reaction rate; [S], substrate concentration.
